# Supplementary material for: Adipocyte lipolysis protects mice against Trypanosoma brucei infection
Source: Nat Microbiol. 2023 Oct 12;8(11):2020–32. doi: 10.1038/s41564-023-01496-7 (PMC10627827; doi:10.1038/s41564-023-01496-7)
Supplement: Supplementary file 1 — Reporting Summary [file 41564_2023_1496_MOESM1_ESM.pdf]

nature portfolio

Corresponding author(s): Luísa M. Figueiredo

Last updated by author(s): Sep 5, 2023

Reporting Summary

Nature Portfolio wishes to improve the reproducibility of the work that we publish. This form provides structure for consistency and transparency in reporting. For further information on Nature Portfolio policies, see our Editorial Policies and the Editorial Policy Checklist.

Statistics

For all statistical analyses, confirm that the following items are present in the figure legend, table legend, main text, or Methods section.

n/a	Confirmed	
☐	☒	The exact sample size (*n*) for each experimental group/condition, given as a discrete number and unit of measurement
☐	☒	A statement on whether measurements were taken from distinct samples or whether the same sample was measured repeatedly
☐	☒	The statistical test(s) used AND whether they are one- or two-sided<br>*Only common tests should be described solely by name; describe more complex techniques in the Methods section.*
☒	☐	A description of all covariates tested
☐	☒	A description of any assumptions or corrections, such as tests of normality and adjustment for multiple comparisons
☐	☒	A full description of the statistical parameters including central tendency (e.g. means) or other basic estimates (e.g. regression coefficient) AND variation (e.g. standard deviation) or associated estimates of uncertainty (e.g. confidence intervals)
☐	☒	For null hypothesis testing, the test statistic (e.g. *F*, *t*, *r*) with confidence intervals, effect sizes, degrees of freedom and *P* value noted<br>*Give P values as exact values whenever suitable.*
☒	☐	For Bayesian analysis, information on the choice of priors and Markov chain Monte Carlo settings
☒	☐	For hierarchical and complex designs, identification of the appropriate level for tests and full reporting of outcomes
☒	☐	Estimates of effect sizes (e.g. Cohen's *d*, Pearson's *r*), indicating how they were calculated

Our web collection on statistics for biologists contains articles on many of the points above.

Software and code

Policy information about availability of computer code

Data collection	LSRFortessa version 6.2 (BD Biosciences), NDP.scan version 1.0 (Hamamatsu Photonics); i-control version 2.0 (Tecan); Minispec Plus version 7.0.0 (Bruker); Quantstudio 5 version 1.5.1.
Data analysis	FlowJo v10.0.7r2 software (Tree Star); GraphPad Prism (version 8.4.3); ImageJ version 1.51h (NIH) with Adiposoft plugin (version 1.16); Quantstudio Design & Analysis software version 2.6

For manuscripts utilizing custom algorithms or software that are central to the research but not yet described in published literature, software must be made available to editors and reviewers. We strongly encourage code deposition in a community repository (e.g. GitHub). See the Nature Portfolio guidelines for submitting code & software for further information.

Data

Policy information about availability of data

All manuscripts must include a data availability statement. This statement should provide the following information, where applicable:

- Accession codes, unique identifiers, or web links for publicly available datasets
- A description of any restrictions on data availability
- For clinical datasets or third party data, please ensure that the statement adheres to our policy

This study does not include large datasets. All data used in this study is available as source data files.

Human research participants

Policy information about studies involving human research participants and Sex and Gender in Research.

Reporting on sex and gender	N/A
Population characteristics	N/A
Recruitment	N/A
Ethics oversight	N/A

Note that full information on the approval of the study protocol must also be provided in the manuscript.

Field-specific reporting

Please select the one below that is the best fit for your research. If you are not sure, read the appropriate sections before making your selection.

☒ Life sciences ☐ Behavioural & social sciences ☐ Ecological, evolutionary & environmental sciences

For a reference copy of the document with all sections, see nature.com/documents/nr-reporting-summary-flat.pdf

Life sciences study design

All studies must disclose on these points even when the disclosure is negative.

Sample size	Sample size was pre-estimated by a power analysis performed on G*Power 3.1 software. Most experiments were performed with at least 4 mice per group and with at least 2 independent experiments.
Data exclusions	Extended Data Figure 4B: Data pertaining to 1 WT mouse that unexpectedly succumbed early to infection was excluded.<br>Extended Data Figure 5A: Excluded two abnormally elevated glycerol measurements noted as outliers through Grubbs' test (1 mouse at day 8 and 1 mouse at day 12 post-infection).
Replication	The experimental findings were reliably reproduced as validated by at least two independent experiments.
Randomization	No randomization. Comparisons between infected and non-infected mice and between Atglfl/fl and AdipoqCre/+ -Atglfl/fl mice were performed using co-housed littermate controls.
Blinding	No blinding was performed in experiments where infected and non-infected mice were compared, as symptoms of infection allow for easy distinction between groups.<br>In experiments comparing infected Atglfl/fl and AdipoqCre/+ -Atglfl/fl mice (Fig. 3-4, 6F and Extended Data Fig.7-8), genotype information was only revealed after mice were euthanized.<br>Investigators were not blinded to group allocation for microscopy acquisitions, however downstream data processing relied on random field acquisition and automated analysis.<br>Acquisition of flow cytometry data, qPCR data and lipolysis data does not involve subjective measurements, reducing the requirement of blinding to group allocation during data collection.

Reporting for specific materials, systems and methods

We require information from authors about some types of materials, experimental systems and methods used in many studies. Here, indicate whether each material, system or method listed is relevant to your study. If you are not sure if a list item applies to your research, read the appropriate section before selecting a response.

Materials & experimental systems

n/a	Involved in the study	
☒	☐	Antibodies
☐	☒	Eukaryotic cell lines
☒	☐	Palaeontology and archaeology
☐	☒	Animals and other organisms
☒	☐	Clinical data
☒	☐	Dual use research of concern

Methods

n/a	Involved in the study	
☒	☐	ChIP-seq
☐	☒	Flow cytometry
☒	☐	MRI-based neuroimaging

Eukaryotic cell lines

Policy information about cell lines and Sex and Gender in Research

Cell line source(s)	EATRO 1125 AnTat1.1E 90-13 from Keith Matthews laboratory (The University of Edinburgh, UK).<br>EATRO 1125 AnTat1.1E 90–13 GFP::PAD1 3'utr cell line from Christian Janzen laboratory (University of Wurzburg, Germany).<br>3T3-L1 (ATCC - CCL-173™. Gaithersburg, MD, USA) from Susana Constantino (University of Lisbon).
Authentication	The cell lines were not authenticated.
Mycoplasma contamination	Cell lines were not tested for mycoplasma contamination.
Commonly misidentified lines<br>(See ICLAC register)	No commonly misidentified lines were used in this study.

Animals and other research organisms

Policy information about studies involving animals; ARRIVE guidelines recommended for reporting animal research, and Sex and Gender in Research

Laboratory animals	Male mice (C57BL/6J WT, Rag2-/-, Tnfa-/-) between 8–12 weeks old.<br>Sex and aged matched AdipoqCre/+ -Atglfl/fl and Atglfl/fl co-housed littermate controls were used aged between 8-20 weeks old.<br>Mice were housed in a Specific-Pathogen-Free barrier facility, under standard laboratory conditions: 21 to 22°C ambient temperature, a 12 h light/12 h dark cycle and 45 to 65% humidity. Chow and water were available ad libitum.
Wild animals	No wild animals were used in this study.
Reporting on sex	Parasite tropism towards the adipose tissue was initially described in male mice. Accordingly, whenever possible male mice were used in this work. Due to limitations in generating sufficient experimental Atglfl/fl and AdipoqCre/+-Atglfl/fl male mice, females were evenly distributed across infected and non-infected groups in figure 3, figure 4C-G, figure 6F and extended data figures 7A and 8. Sample sizes in this study are insufficient to perform post hoc analyses based on sex.
Field-collected samples	No field-collected samples were used in this study.
Ethics oversight	Animal experiments were performed according to EU regulations and approved by the Órgão Responsável pelo Bem-estar Animal (ORBEA) of Instituto de Medicina Molecular João Lobo Antunes and the competent authority Direcção Geral de Alimentação e Veterinária (licenses: 018889\2016 and 017549\2021).

Note that full information on the approval of the study protocol must also be provided in the manuscript.

Flow Cytometry

Plots

Confirm that:

☒ The axis labels state the marker and fluorochrome used (e.g. CD4-FITC).

☒ The axis scales are clearly visible. Include numbers along axes only for bottom left plot of group (a 'group' is an analysis of identical markers).

☒ All plots are contour plots with outliers or pseudocolor plots.

☒ A numerical value for number of cells or percentage (with statistics) is provided.

Methodology

Sample preparation	AnTat1.1E GFP::PAD1 3'utr reporter parasites isolated from adipose tissue by gentle agitation in culture medium or from 3T3-L1 co-cultures by harvesting culture supernatants . These cells were then fixed with either formaldehyde or ethanol and stained with Hoechst 33342 or propidium iodide and then filtered prior to acquisition.<br>AnTat1.1E parasites in axenic cultures were stained with propidium iodide and filtered prior to acquisition.<br>Splenocytes were obtained from non-infected WT mice, cell suspensions obtained through mechanical desegregation and subjected to red blood cell lysis. Splenocytes were then used in axenic cultures, stained with propidium iodide and filtered prior to acquisition.<br>3T3-L1 pre-adipocytes were cultivated to 70-80% confluence, passaged using trypsin and used in axenic cultures followed by staining with propidium iodide and filtered prior to acquisition.
Instrument	LSRFortessa (BD Biosciences)
Software	FACSDiva software version 6.2 (BD Biosciences) for data acquisition and FlowJo software version 10.0.7r2 (Tree Star) for analysis.
Cell population abundance	No cell sorting was performed in this study.

Gating strategy	After excluding doublets through SSC-W and SSC-A gating, stumpy forms were identified as Hoescht intermediate and PAD1 positive.<br>Non-viable parasites were identified based on positive propidium iodide signal.<br>Boundaries between positive and negative populations were determined using non-stained controls.<br>Assignment of cell cycle stages was performed based on visual of propidium iodide histogram distribution using a linear scale.

☒ Tick this box to confirm that a figure exemplifying the gating strategy is provided in the Supplementary Information.
